# Research Considerations for the Use of Publicly Available Documentary Films to Study Refugee Family Therapy

**DOI:** 10.3390/ijerph23020265

**Published:** 2026-02-20

**Authors:** Charity Mokgaetji Somo

**Affiliations:** Department of Psychology of Education, The University of South Africa, Pretoria 0002, South Africa; esomocm@unisa.ac.za

**Keywords:** family therapy, documentary films, qualitative conventional content analysis (QCCA), qualitative research, refugee family trauma, video material

## Abstract

Scholars in family therapy are increasingly calling for family-centered interventions for trauma-affected refugees, as many trauma-informed therapies favor individual models of treatment. Research contributes to the study and implementation of family-centered care models. However, for methodological reasons, research on family therapy with displaced populations is limited. In response to scholars’ call, this paper argues for the use of documentary film as qualitative research data in refugee family therapy research. Documentary films have historically been used in the social sciences to examine people’s lived experiences and to address data gaps in hard-to-reach populations. This paper outlines key methodological considerations inherent in research with refugee populations, including challenges related to recruitment and retention, language and cultural barriers, insecure and unstable living conditions affecting participants, research design constraints, and ethical complexities. It then discusses how the use of documentary film can help mitigate these challenges through careful epistemological positioning, research design, data selection and analysis strategies, and attention to ethical and research trustworthiness considerations. By doing so, the paper contributes to the development of qualitative research skills necessary for studying refugee family well-being and supporting the growth of family-centered therapeutic approaches.

## 1. Introduction

The current global refugee crisis is not abating, with the number of displaced people doubling in just a decade [1]. At the end of 2024, approximately 123.2 million people worldwide had to flee their homes and communities due to war, human rights violations, and persecution [1]. Catastrophic events such as war result in physical and psychological trauma for the displaced. Research shows that refugees suffer from high rates of mental health disorders, including depression, anxiety, post-traumatic stress disorder (PTSD), and suicidal behaviors [2,3,4]. Refugee families often experience migration trauma events as a collective. During pre-migration, families can encounter war atrocities such as the destruction of the family home, debilitating injuries, and the death of family members [5]. The migration journey to safety poses several vulnerabilities, including abuse by smugglers and human traffickers who target women and children, and separate families through kidnapping [6]. Safety often means living in refugee camps, which are often inadequate living environments, marked by conditions that undermine dignity, safety, and well-being [5]. Families who are granted asylum in third countries have to grapple with resettlement stress. For example, learning the local language, finding employment, and adapting to the local culture can improve chances of adaptation, but it can initially take a toll on the family system [7]. Such migratory stressors and traumatic events can lead to several family-level trauma outcomes [8].

Despite the challenges of forced migration, refugee families can find meaningful ways to rebuild their lives. Resources for positive resettlement include family cohesion, community connections, and cross-cultural participation [9]. Community acceptance and support from locals and refugee networks significantly predict family health and positive mental health [9]. Family therapy also plays an important role in fostering resilience among refugee families [10]. Scholars in family therapy are increasingly calling for family-centered interventions for trauma-affected refugees, as many trauma-informed therapies favor individual models of treatment. Research contributes to the study and implementation of family-centered care models. However, research on family therapy with displaced populations is limited [11]. In response to scholars’ call, this paper argues for the use of publicly available documentary film as a viable data source in refugee family therapy research. Documentary films have historically been used in the social sciences to examine people’s lived experiences and to address research gaps in hard-to-reach populations. This paper aims to reflect on the use of publicly available documentary film data in the study of systemic therapy for refugees. The reflection is based on the research methodologies first developed and applied in the author’s PhD dissertation [12]. The methodology is selectively drawn on here to advance methodological discussion of the use of documentary film in the study of refugee well-being. The PhD study was empirically oriented, examining how family therapists conducted therapy with refugee families. In contrast, this paper foregrounds and critically reflects on the key methodological considerations in the use of available documentary films in refugee family therapy research. It presents a methodological re-analysis that contributes to research on refugee family well-being and supports the development of family-centered therapeutic approaches.

## 2. Background

The following discussion outlines the challenges in research on refugee systemic therapy and explores the potential of using publicly available documentaries to address gaps in refugee family therapy research.

### 2.1. Research Methodology in Refugee Family Therapy

Systematic reviews have reported only a small number of family-based intervention studies and even fewer that evaluate the effectiveness of systemic treatments [11]. Scholars report that the following methodological factors can complicate and delay studies in this field [11,13,14]:

1. Recruitment and retention. Participant recruitment can be challenging because many refugees have limited access to formal mental health services, where participants are typically identified. Community-based recruitment frequently depends on approval from gatekeepers such as community elders, whose cooperation is influenced by the community’s familiarity with psychotherapy and the level of trust established with researchers [13,14]. These constraints can result in small sample sizes and slow data collection. Retention is further challenged by logistical barriers, including limited access to private data collection spaces, financial and transportation constraints, and ongoing language and cultural mismatches between participants and researchers. High dropout rates should be understood within this broader ecological context rather than interpreted solely as methodological failure [13]. However, these logistical issues can lead to limited research data and prolonged data collection. Publicly available film documentaries depict therapeutic encounters that have already taken place. Participants in these films have been recruited and have participated in the family therapy intervention. This reduces reliance on prolonged participant recruitment and sustained participant engagement over time.
2. Language and cultural barriers. Refugee research is commonly conducted in host countries where linguistic and cultural contexts differ from those of participants. This necessitates the use of interpreters and cultural brokers, which increases costs and time demands [11]. Furthermore, the use of cultural and language brokers can risk meaning loss or inconsistent data collection. Researchers highlight that many assessment tools are developed and validated in Western populations, raising concerns about cultural validity, symptom conceptualization, and the appropriateness of standardized measures across cultures [13]. Documentary films are typically recorded in participants’ local languages and often include English subtitles or voice-over, making the material accessible to researchers conducting analysis in English. As a result, researchers can engage with the data without the additional demands often associated with managing cross-linguistic and cross-cultural dynamics during in-person interviews or primary data collection. At the same time, researchers can still consider how participants’ cultural and contextual circumstances may influence the findings when interpreting the data.
3. Insecure and unstable living conditions. Many refugees live in temporary or resource-insecure environments and depend on external agencies for housing, legal status, and basic needs. Such instability creates uncertainty about research participation, particularly when families fear consequences related to confidentiality, immigration status, or access to services [13]. Ongoing stressors in living conditions—including financial insecurity and legal procedures—can also reduce participants’ capacity to prioritize research involvement and may affect informed consent and engagement. Researchers using publicly available films can conduct analysis without repeatedly engaging participants in data collection, while still upholding ethical standards and considering how the context and experiences captured in the recordings may influence interpretation.
4. Research design constraints. Key elements of randomized controlled trials—such as control groups, standardized treatment dosages, and rigid protocols—often limit feasibility in refugee contexts and reduce the applicability of findings to real-world practice [10]. For this reason, it is often difficult to assess the effectiveness of systemic treatments for displaced populations. The use of documentary films as data can help mitigate research design constraints by providing rich qualitative data in contexts where randomized controlled trials are often impractical. By making therapeutic encounters accessible for analysis, documentary-based research enables the study of systemic interventions that might otherwise be difficult to examine. This supports increased research activity and fosters informed scholarly discourse on the effectiveness and application of these therapies in real-world refugee contexts.
5. Ethical complexities. Researchers working with refugee populations frequently encounter ethical tensions, including overinvolvement, role strain, and the need to balance methodological rigor with responsiveness to participants’ immediate social and psychological needs [13]. Addressing these ethical obligations often necessitates protocol adaptations that, while ethically appropriate, may further complicate replication and methodological consistency. While the use of publicly available data can reduce some of these challenges, researchers remain ethically responsible for respecting participants’ dignity [15], exercising reflexivity, and carefully considering researcher positionality.

Scholars argue that these constraints have necessitated a shift toward more practical and adaptable frameworks for conducting psychotherapy research with displaced populations [14]. Researchers advocate for the inclusion of qualitative approaches—such as ethnographic methods—in future studies [11]. Integrating these methods is considered essential to strengthening research on family-based interventions for displaced populations. This paper advocates the use of documentary films as a qualitative data source in studies of refugee family therapy. The paper also aims to provide justification for the use of documentary film in refugee therapy scholarship. While the methodological factors identified in the literature significantly complicate and delay refugee family therapy research, they also highlight the need for more flexible and context-sensitive research approaches. The use of documentary film as a qualitative data source does not eliminate challenges associated with data collection in refugee studies. It offers a methodological framework that can mitigate their impact by reducing reliance on direct participant recruitment, minimizing cross-cultural data collection demands, accommodating unstable living conditions, and enabling ethically responsive research design. By shifting the unit of analysis from in-person data collection to existing filmed therapeutic encounters, documentary-based inquiry provides a practical and ethically viable alternative for studying systemic family therapy with displaced populations.

This paper will contribute to research on family therapy with refugee populations. To the author’s knowledge, no research has yet been conducted on the use of documentary films to study family therapy with refugee populations. One scholar noted that as early as the mid-1950s, prolific researchers, such as Gregory Bateson, Jürgen Reusch, and Weldon Keys, used the new technology of that period to film and study family therapy [16]. Due to the availability of video material and increased efficiency in collecting, analyzing, sharing, and archiving it, scholars are now incorporating video material into the learning process in many scientific disciplines [17]. As such, video material (including archival formats) may be a viable alternative to data collection in studies of psychotherapy with refugees.

### 2.2. Visual Qualitative Research Methodologies

In the social sciences, video documentaries are films that depict people’s lived experiences regarding social concerns and political phenomena [18]. Visual methods are an emerging, novel approach to qualitative research grounded in traditional ethnographic methods [19]. In ethnographic studies, visual methods have been used to study human interactions in their natural context [20]. Ethnographers examine these interactions as they occur, and they elaborate on the nuances of human relationships within the organic environment [20]. In the social sciences, documentary filmmakers are interested in showing the lived experiences of humans in unique and/or challenging circumstances, such as the refugee crisis (see, for example, Hiltunen [21]). Film and video content have always been important in showing the lived experiences of refugee families. Journalists and ordinary people use photography and video to show the devastation caused by war and its consequences for displaced people. Technology has made it possible for these experiences to be widely publicized. For instance, when the war in Ukraine started, thousands of people around the globe watched real-life war combat, bombings, and refugee migration on several social media platforms and video streaming services daily for several weeks. The experiences filmed and shown on video streaming services constitute data and can be effectively used in refugee family studies.

The benefits of visual data also apply to documentary film data. Firstly, documentary films are a novel way to study participants who are difficult to include in research studies, such as refugee families [19]. Ethnographic filmmakers have always used film to study cultures and the lived experiences of populations. Studies on forced migration can learn from ethnographic methodologies to meet research needs. This study shares the growing interest in using visual methods in qualitative data analysis, as the researcher observed that these methods can yield thick data and strengthen research efforts in refugee family therapy. Secondly, documentary films add a distinct dimension to research [19] by integrating participant observation, participant narratives, and contextual information within a single medium. For example, in analyzing a documentary film on refugee family therapy, the researcher can observe multiple family members’ verbal (narratives) and nonverbal interactions (emotional and behavioral reactions) simultaneously. Thirdly, documentary films depicting the refugee crisis are often filmed within the context of refugees’ real experiences, for example, in refugee camps. While the experiences captured in videos may seem emotionally charged and biased toward desperation, it can be argued that war produces devastation and desperation. Therefore, there is nothing else but destitution and desperation to be shown. Through these films, the research becomes contextually and culturally sensitive [19]. Lastly, documentary films can be used to engage public opinion and activism [20,22]. They are now also being used to research and disseminate knowledge on refugee experiences (see, for example, Hiltunen; Nisbet & Aufderheide; Zhang, 2018 [21,22,23]). Hiltunen analyzed documentary films depicting the migration crisis in the Middle East and sub-Saharan Africa [21]. In such films, filmmakers emphasize respecting their subjects as individuals and giving them the chance to tell their own stories [21]. Such a perspective is pivotal in qualitative research, where researchers aim to explore participants’ lived experiences through their subjective perceptions.

These benefits demonstrate the potential of visual methods, such as documentary films, to close the research gaps in refugee family well-being research. The next section provides an example to demonstrate how publicly available documentary films can be used to study refugee family therapy. The discussion highlights key research considerations for studying refugee family therapy, including epistemological positioning, methodological approach, data collection, data analysis, ethics, and research trustworthiness.

## 3. Methodological Considerations

As stated earlier, this paper is a secondary analysis of the author’s PhD study [12], which utilized documentary films to explore how relational therapists provide family therapy for refugee families. The exploration was guided by two research questions: (1) How do scholars articulate and conceptualize refugee relational psychological trauma? (2) What are the important considerations when providing systemic therapy to refugee families? The findings of the PhD study are presented in the original study [12]. The next section provides an example to demonstrate how publicly available documentary films can be used to study refugee family therapy. The example offers a methodological framework that can lessen the impact of methodological challenges in refugee family therapy research. In this example, the unit of analysis shifted from data collected through conventional in-person methods to publicly available film documentaries as the primary data source. The discussion highlights key research factors for studying refugee family therapy using film data, including epistemological positioning, methodological approach, data collection, data analysis, ethics, and research trustworthiness.

### 3.1. Research Epistemology and Approach

In documentary studies, family therapy is viewed through the filmmaker’s lens. This then begs the question of how filmmaking shapes knowledge production. Drawing on Hiltunen’s observation that documentary filmmakers often seek to treat participants as competent subjects who speak for themselves [21], knowledge can be regarded as co-constructed by participants, filmmakers, and the researcher. Knowledge is constructed from what is portrayed through the camera lens, what is communicated by the film subjects, and the researcher’s analysis. Scholars assert that knowledge in documentary films is co-produced rather than discovered [24]. Studying therapy through a filmmaker’s lens, therefore, entails acknowledging that knowledge emerges from participants’ narrated experiences and is co-constructed with filmmakers through cinematic choices. In this respect, documentary-based inquiry aligns closely with constructivist qualitative approaches, which frame reality as multiple, situated, and produced through social interaction.

A qualitative approach aims to explore participants’ lived experiences through audio, visual, and textual representations [25]. This approach is well-suited to documentary film research and can be used to examine the experiences of family therapists conducting systemic therapy with displaced populations. An important part of qualitative research is to explore experiences through participants’ narratives and subjective perceptions [26]. In the documentaries, participants share their personal experiences of displacement in their own words and from their perspective, explaining the meanings they have drawn from them. An additional strength of documentary films is their ability to show participants actively living out the events or experiences under study. In film documentaries, the researcher can observe both the therapist and the family during family therapy and analyze the therapeutic situation from multiple perspectives. Qualitative research also emphasizes the context in which the experiences occurred [26]. Context plays a pivotal role in interpreting participants’ lived experiences. Documentary films are effective in qualitative research because they seek to richly describe subjects’ demographic and contextual profiles and personal stories [21]. The thick description of participant context, demographics, and lived experiences is a core characteristic of qualitative research. The unique feature of documentary films is that they provide visual evidence of the stories participants tell, thereby enabling methodological triangulation within a single setting. In summary, the qualitative use of documentary films offers a rigorous and contextually grounded methodology for examining systemic family therapy with displaced populations. By integrating participants’ narratives, observed interactions, and rich contextual detail, documentary films enable a thick, multi-perspectival understanding of lived experience while supporting methodological triangulation. This approach not only aligns closely with the epistemological aims of qualitative research but also enhances the depth, credibility, and interpretive potential of studies focused on complex therapeutic and displacement contexts.

### 3.2. Data Collection

Defining data. Documentary films are a genre that seeks to represent aspects of reality by translating real people, places, and events onto the screen through a creative treatment of actuality [27]. When selecting documentary film data from public platforms, researchers should select those that are information-rich and relevant to the research question. Purposive sampling can be used to identify film data that aligns with the research question. This approach involves selecting videos that directly address the study’s aims and involves an exhaustive search to identify available material across platforms and formats [12]. Conducting such an initial search is important for establishing the full range of potential data before applying predefined inclusion criteria.

Inclusion criteria. Inclusion and exclusion criteria are particularly valuable when working with domains characterized by abundant content but uneven relevance, such as video material on refugee migration. Inclusion criteria can guide the researcher to select a credible sample. To include films that provide rich and relevant data, the following inclusion and exclusion criteria can be regarded:

- Publicly available videos. When selecting videos, researchers should consider free options so that anyone can assess their transferability without spending money. For example, videos from professional psychology databases cost a fee, making them difficult to access.
- Therapist credentials. Therapists featured in the documentary films need to disclose that they are registered mental health professionals in their country of practice. Publicly available videos can be uploaded by anyone. Researchers can verify therapists’ credentials on official websites where they are listed.
- Trauma-informed interventions. There are videos on refugee migration that discuss the refugee crisis without a discussion on psychotherapy. Researchers should ensure that videos feature trauma-informed interventions. This can be determined by watching each video in full.
- Current materials. The migration crisis has been going on since the beginning of time. Some library archives have videos dating back decades. It is best to include videos recorded within the past recent years to ensure contextual relevance.
- Ethical depiction. Researchers should take care to protect the dignity of refugee families depicted in the video materials. While a war-related refugee crisis can be dreadful, all people deserve to be presented in a manner that exemplifies their human dignity.

Example of application of inclusion criteria from Somo [12]: Video materials depicting refugees engaging in family therapy were identified from the following sources:

1. Professional psychology databases were searched for video material, including psychotherapy.net, the APA Psychotherapy Video Series, counseling and therapy videos, and psychotherapy videos and DVDs. Professional platforms are an effective way to ensure that therapies are conducted by licensed therapists who use trauma-informed therapies. Professional platforms also tend to have recently documented videos.
2. Academic libraries, including the University of Georgia Libraries (e.g., Films on Demand, which specializes in educational films). Academic libraries allow cost-free access to video materials. Libraries also permit the free interlending of video materials from other public platforms. Library systems tend to offer video materials dating back decades.
3. Public internet video platforms, such as YouTube and Vimeo. Ultimately, none of the videos found on these platforms met the inclusion criteria. Videos on these platforms included themes of refugee migration (e.g., life in refugee camps, refugee voyages). However, they did not depict any stories related to psychotherapy. Additionally, many videos on public platforms are very short (often less than 10 min long) and present a snapshot of the story, which is not conducive to meaningful analysis.

Exclusion Criteria. An exclusion criterion further delimits the selection of materials that provide relevant information for the research question. For example, Somo [12] used the following criteria to further exclude irrelevant films from the study:

- Videos that featured refugee health without discussing mental health,
- Videos that discussed refugee war experiences but not refugee mental health,
- Videos that discuss refugee migration but not refugee mental health outcomes.

In the end, five videos met the inclusion criteria and were included for analysis. The five videos fall into two types: three family therapy documentaries and two educational videos on mental health services for refugee families impacted by war trauma. Videos relating to psychotherapy fell within ethical boundaries; therefore, no videos were excluded for ethical reasons. Table 1 presents an overview of the final video materials and the rationale for their inclusion.

### 3.3. Data Analysis

**Figure 1:**
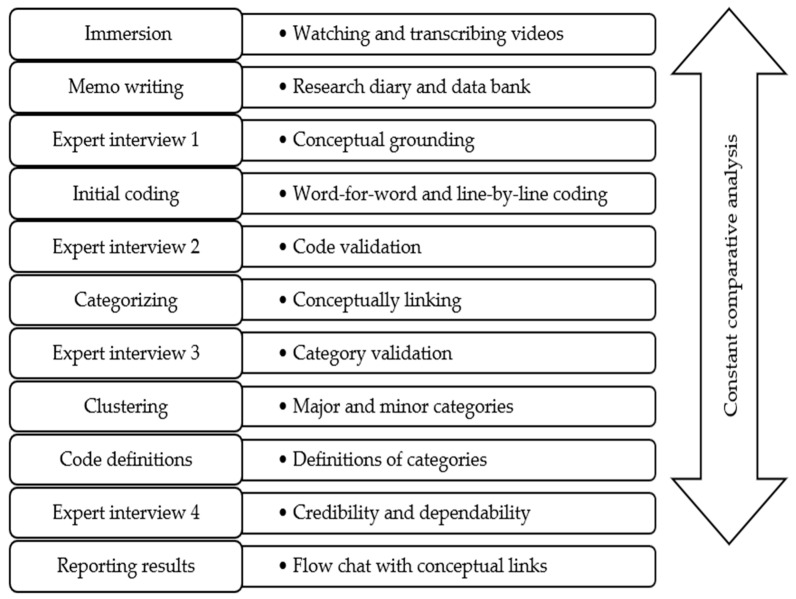
Qualitative Conventional Content Analysis of Documentary Films.

According to Fouché et al. [33], the primary criticism of utilizing non-textual data lies in the difficulties associated with its analysis. Documentary films can be analyzed using a systematic research protocol that is aligned with the qualitative research approach. Borish et al. [34] developed a video-based qualitative protocol to analyze documentary films. They report that the approach is grounded in the understanding that video interviews created through documentary filmmaking constitute qualitative data and can be analyzed using recognized qualitative methods. Qualitative conventional content analysis (QCCA) is a research approach that systematically codes text data and identifies themes or patterns to enable subjective interpretation of the content [25]. QCCA is especially relevant to analyzing documentary films as it offers methodological procedures to analyze videos and transcripts [25]. In the study that provides context for this particular paper, QCCA was employed as a data analysis strategy for documentary film data. Figure 1 depicts an example of how QCCA can be used data as an analysis procedure for documentary films in refugee studies. While Hsieh and Shannon [25] propose a seven-step process, the procedure in this example was extended to 12 steps to incorporate methodological triangulation and constant comparative methodology. Fouché et al. [33] suggest using additional methods to triangulate visual material to enrich the data; therefore, expert consultations were added as a means of data triangulation. The constant comparative method by Charmaz [35] was used to compare codes as they emerged from the analysis. The section below explicates the analysis procedure.

1. Immersion. Immersion in video material enables the researcher to understand the data as a whole [25]. This step is essential for video data, where the researcher analyzes it multidimensionally. Immersion can occur in two ways: repeatedly watching video materials and transcribing them.
2. Constant comparative analysis. The constant comparative method compares codes across different levels of analysis to find similarities and differences [35]. This step is not part of the QCCA strategy but can be added to enrich the data analysis process. It ensures that codes emerge from the data through interactive examination and constant comparison of codes.
3. Memo writing. Memo writing is for reflective and analytical purposes. Memos are critical for the reflections on the contextual background shown in the videos. These details contribute towards coding, categorizing, and defining the emergent themes. Memos also document conceptual developments as they emerge from integrating expert feedback.
4. Expert interview 1: As stated by Fouché et al. [33], triangulation methods are important when using video materials to enhance the trustworthiness of findings. Topic expert interviews can serve as a source of triangulation. Researchers can interview experts at different stages of the analysis to confirm emerging findings. In this example, expert 1 was consulted to identify key sensitizing concepts to compensate for the limited literature on the topic.
5. Initial coding. Initial coding is conducted through word-by-word coding (to identify keywords) and line-by-line coding (to interpret their meanings).
6. Expert interview 2: Code validation. Following the initial coding, a second expert can be consulted. The expert may provide feedback on salient codes in the topic and on underrepresented codes in the existing literature.
7. Categorization. Categorizing is conceptually linking codes and organizing them into meaningful units [25]. This inductive process preserves the emergent nature of findings.
8. Expert interview 3: Category validation. An expert can be consulted to review emerging categories. The number of experts consulted is dependent on the researchers’ empirical confidence in emerging findings. It is recommended that experts be consulted until data has reached saturation and the methodology has been validated.
9. Clustering. Categories developed in step 7 can be organized into theoretical clusters. Clustering results in the formation of final major and minor categories.
10. Defining categories and subcategories. Definitions for each category are developed using the memos. Memos contain conceptual links between the different stages of coding and the contextual information noted in step one of this procedure.
11. Expert interview 4. Methodological experts can be consulted to assess methodological rigor. While the field of using documentary films in qualitative research has recently attracted attention, the method remains relatively new. Therefore, method experts can improve credibility and dependability through evaluating the data trail.
12. Reporting of findings. Hsieh and Shannon [25] suggest presenting results in a flowchart that shows the conceptual links among codes, categories, and emergent themes. Some researchers using documentary data (e.g., Borish et al. [34]) include still photos to contextualize their findings. Reporting through flowcharts and photographs is consistent with qualitative designs.

### 3.4. Ethical Considerations and Research Trustworthiness

Studies using publicly available data can be exempt from institutional ethics approval, as they involve the use of secondary data. Nevertheless, research using publicly available data should be carried out for participants’ benefit, to use the data for good and to provide a just and fair representation of participants’ lived experiences [14].

The well-established criteria of rigor—credibility, dependability, transferability, and confirmability [36,37]—provide a helpful framework for enhancing methodological rigor in studies that incorporate documentary film [24]. Credibility refers to the extent to which research findings are an accurate and trustworthy representation of the data as interpreted by the researcher [38]. In documentary film research, credibility can be strengthened through the use of multiple, complementary strategies. Researchers may enhance analytical rigor by establishing a clear audit trail that demonstrates alignment between research questions, data sources, and analytic decisions. Engaging a research advisory group to review analytic processes can further support credibility by ensuring transparency and methodological coherence. Triangulation can be achieved through consultation with subject matter experts relevant to the study context, such as clinicians or practitioners working with refugee families. These experts can be invited to review emerging codes and interpretations at successive stages of analysis, offering critical feedback on the plausibility, coherence, and validity of analytic claims.

Dependability refers to the consistency of the research process and the extent to which it can be repeated across similar contexts and participant groups [38]. A detailed audit trail can also ensure dependability [24,38]. Detailed methodological documentation enables other researchers to follow, evaluate, and potentially replicate the study under comparable conditions. Periodic peer review of the research process can further support structural corroboration and methodological consistency. Working in a research team and holding regular discussions among researchers can ensure that research data is informed by adherence to research protocols [24], which enhances both dependability and confirmability.

Confirmability addresses the degree to which findings are grounded in the data rather than shaped by researcher bias or presuppositions [38]. Researchers can enhance confirmability by practicing sustained reflexivity throughout the research process. Reflexive journaling and analytic memo writing can be used to document assumptions, emotional responses, and interpretive decisions, thereby making the researcher’s influence on the analysis visible and open to scrutiny.

Transferability concerns the extent to which findings may apply to other contexts or populations [38]. To facilitate assessments of transferability, researchers should provide rich, contextualized descriptions of the documentary materials analyzed, including details on the setting, participants, and thematic focus. Such detail allows readers to determine the relevance of the findings to their own research or practice contexts. A central strength of publicly available documentary films is their accessibility, which allows readers to view the material directly and independently assess the transferability of the findings to their own contexts.

## 4. Discussion

The literature highlights persistent methodological challenges in refugee family therapy research—including recruitment and retention difficulties, language and cultural barriers, insecure and unstable living conditions, research design constraints, and ethical complexities—that continue to limit the feasibility and rigor of conventional empirical approaches. This paper responds to these challenges by advancing documentary film as a qualitative data source grounded in a constructivist epistemology, demonstrating how careful research design, purposive data selection, systematic qualitative analysis, and attention to ethics and trustworthiness can offer a contextually responsive methodological framework for studying systemic family therapy with displaced populations.

Like any other research methodology, the use of documentaries in academic research has received both praise and criticism [27]. Using documentary film to study refugee family therapy has its own strengths and limitations, and they are discussed next.

### 4.1. Strengths of Documentary Films

The strengths of using documentary films on refugee therapy include:

1. Research accessibility under challenging contexts. The researcher can source data in displacement where research and the therapy process are difficult to document [10].
2. Authentic representation of lived experiences [20]. Refugee documentary films are used to depict the family and mental health realities of refugee families. Researchers can use these lived experiences to inform family therapy interventions.
3. Multidimensional analysis of the therapy process. Visual methods capture rich multidimensional data [19]. In therapeutic spaces, the researcher can analyze interactions among the therapist and the family, as well as among family members. They can also analyze nonverbal interactions, such as emotions, gestures, and behavioral cues.
4. Perspectives from multiple participants. Aufderheide [39] explains that documentary films are powerful narrative tools representing diverse voices and participant perspectives, often allowing underrepresented or marginalized groups to articulate their experiences.
5. Contextual environmental analysis. Documentary films allow for analyzing the participants’ social, political, and cultural contexts as they relate their experiences.

The use of documentary film within academic research has been widely endorsed and critiqued, reflecting ongoing debates about its epistemological and methodological status [27].

### 4.2. Limitations of Documentary Films

While this paper asserts that documentary films can bridge gaps in refugee family therapy scholarship, it acknowledges limitations. Firstly, documentary films are not objective. Qualitative research does not claim to be objective. It is the interpretation of subjective accounts of participants’ lives and/or the meaning they have constructed from their life stories. Documentary films are considered authentic representations of events in the lives of the observed [20]. There is typically no written or performative script for recordings, and participants are filmed engaging in activities as they ordinarily occur. Despite this, critics of documentary filmmaking argue that these representations may oversimplify complex social realities and involve varying degrees of selection and emphasis [27]. Because filmmakers retain control over what is recorded, edited, and ultimately presented, documentaries may risk offering partial or unfair representations of lived realities, raising questions about the accuracy and balance of the messages conveyed. In some cases, the filmmaker’s interpretive standpoint may exaggerate particular themes for narrative or affective impact [27]. For these reasons, researchers should consider engaging topic experts to examine emerging themes in the data analysis. Researchers should also compare their findings with current literature and theoretical perspectives.

Secondly, documentary films are subject to a camera effect. When individuals are filmed, they may consciously or unconsciously present aspects of themselves or their experiences that they consider personally, socially, or politically significant. In documentaries focusing on refugees, this self-presentation may be shaped by political agendas, social advocacy aims, or funding priorities. While researchers should critically assess the implications of the camera effect, it is important to note that similar dynamics can arise in audio-recorded or interview-based research once participants are aware that they are being recorded.

Thirdly, an additional limitation concerns the challenge of documenting a clearly defined, methodical research process [40]. Researchers can address this limitation by explicitly articulating how video materials were collected, analyzed, and interpreted [40]. Researchers should also acknowledge methodological issues when reporting their findings and clearly articulate the limitations of documentary film data.

Documentary films offer significant methodological and conceptual strengths for research on family well-being by enabling rich, contextualized, and embodied accounts of lived experience. Their capacity to capture affective, relational, and environmental dimensions of refugee lives provides insights that are particularly compelling. At the same time, the use of documentary film as research data introduces important limitations, including subjectivity in representation, the influence of the camera on participants’ behavior, the filmmaker’s interpretive authority, and challenges related to transparency and methodological documentation. These limitations do not invalidate documentary film as a research method but underscore the need for critical reflexivity, methodological rigor, and explicit reporting of analytical processes.

## 5. Conclusions

The methodological challenges that complicate refugee family therapy research underline the need for flexible and context-sensitive research approaches. Using video material to study family therapy with refugee families can fill the methodological gaps in the literature. Research systematically collects data to investigate and report findings on a phenomenon. Using documentary films in research requires systematic data collection, analysis, and reporting to ensure findings are consistent with research trustworthiness. This paper adds to the research on refugee family therapy by providing a methodological rationale for using documentary films and outlining practical steps for data collection and analysis of video material. It is hoped that this paper will enhance the research skills needed to study the well-being of refugee families.

## Figures and Tables

**Table 1:** Documentary films on refugee family therapy.

Video Reference	Video Summary
[28]	This documentary film was included in the study because the therapists discuss the violence and trauma experiences of refugee families pre- and post-migration. The therapists also provide examples of systemic ways to address the needs of families who have been through war-related violence.
[29]	This video depicts the psychological, personal, and relational effects of trauma on individuals and families. It also shows how traumatic stress is experienced by people who have gone through different traumatic events. In these videos, symptoms of traumatic stress are discussed. The therapists also discuss interventions with refugee individuals and families.
[30]	This material was selected because it explains the importance of support systems for people who have gone through trauma. Support systems have also been a source of resilience for refugee families.
[31]	The video examines and discusses trauma events that lead to displacement and presents a systemic and culturally relevant model for addressing refugee family trauma.
[32]	In this video, the contextual factors that cause post-migration stress and inhibit positive mental health among refugee families are highlighted. The use of contextually and culturally relevant therapies for refugee families is explored.

## Data Availability

The data used in this paper are publicly available video materials accessible via the links provided in the reference list. Access to these materials requires payment, and in some cases, readers must create login credentials prior to purchase.
